# Comparative Analysis of the *aquaporin* Gene Family in 12 Fish Species

**DOI:** 10.3390/ani9050233

**Published:** 2019-05-13

**Authors:** Jun Cao, Feng Shi

**Affiliations:** 1 Institute of Life Sciences, Jiangsu University, Zhenjiang 212013, China; 2 Corporation of Lvdou Biopharmaceuticals, Binzhou 256600, China; fshi456@163.com

**Keywords:** intragenic recombination, evolution, selective pressure, expression, organophosphorus pesticide

## Abstract

**Simple Summary:**

Aquaporins (Aqps) are a group of membrane proteins. In this study, 166 *Aqp* genes were identified in 12 fish species. Gene organization, motif distribution, recombination, and selection pressure were performed to investigate their evolutionary characteristics. In addition, expression profiles of *Aqps* were also examined under pathogens infection and organophosphorus pesticide stress. This study will provide a useful reference for further functional study.

**Abstract:**

Aquaporins (Aqps) are a class of water channel proteins that play key roles in many physiological functions and cellular processes. Here, we analyzed 166 putative *Aqp* genes in 12 fish species and divided them into four groups. Gene organization and motif distribution analyses suggested potentially conserved functions in each group. Several recombination events were identified in some members, which accelerate their divergence in evolution. Furthermore, a few positive selection sites were identified, and mutations at these sites could alter the stability of Aqp proteins. In addition, expression profiles of some *Aqp* genes under pathogen infection and organophosphorus pesticide stress were also investigated. The result implied that several *Aqp* genes may affect different immune responses and osmoregulation. This study provides a comparative analysis of the fish *Aqp* gene family to facilitate further functional analyses.

## 1. Introduction

Aquaporins (Aqps) are a set of small (26–34 kDa) membrane proteins that specifically transport water, glycerol, ammonia, and urea across cell membranes [1,2]. They typically consist of six transmembrane alpha-helices surrounding a central water-transporting pore and two loops forming hemi-helices. Aqps typically form as a homo-tetramer embedded in the membrane, and each monomer acts independently as a single channel [3,4]. Aqps contain two conservative NPA (Asn—Pro—Ala) motifs that form short hydrophobic helices, which constitute the first filter region in substrate selectivity [3]. Another filter region is located in the extracellular channel and consists of several aromatic amino acids and an Arg residue (ar/R). This region creates the narrowest section and determines substrate specificity of this channel [5,6]. These conserved motifs exist in most members of the Aqp family and create two restriction sites that allow water and some other small solutes to pass [7]. In addition, the least conserved N- and C-terminal domains of the Aqp proteins are also associated with channel gating, intracellular localization, and membrane trafficking and assembly [8,9,10].

Aqps are classed into four major subgroups in eukaryotic organisms based on their sequence characteristics: (1) classical aquaporins (Aqp0, 1, 2, 4, and 5), which only permeate water; (2) aquaglyceroporins (Aqp3, 7, 9, and 10), which permeate glycerol and urea in addition to water; (3) Aqp8-type of aquaammoniaporins (Aqp6 and 8), which present low water permeability and different phylogenetics from the others; and (4) unorthodox aquaporins (Aqp11 and 12), which have highly deviated NPA motifs and intracellular locations [11,12,13]. Aqps are expressed in a wide range of tissues. For example, fish *Aqp1aa*, *-3a*, and *-12* are expressed ubiquitously in all examined tissues, indicating constitutive roles of transporting fluids between cells [14]. Conversely, the expression profiles of zebrafish *Aqp0a* and *-0b* are more restricted in the lens, suggesting that they are necessary for normal lens development [15]. Some members of the zebrafish *Aqp8* gene family (*Aqp8a.1*, *Aqp8a.2,* and *Aqp8b*) exhibit different spatiotemporal expression patterns during early embryonic stages, implying that they play distinct roles during embryonic development by regulating organ-specific intracellular water balance [16]. Moreover, the cellular functions of Aqps are also regulated by post-translational modifications (PTMs), e.g., acetylation, phosphorylation, methylation, ubiquitination, and glycosylation. PTMs adjust localization, stability, and activity by altering protein conformation [17]. In teleosts, phosphorylated Aqp8b inserts into the inner mitochondrial membrane of spermatozoa to facilitate hydrogen peroxide efflux from this compartment, which plays a key detoxification function under hypertonic conditions [18]. The phosphorylated Ser256 residue of Aqp2 also increases water migration [19]. In addition, reduced protein ubiquitination and increased protein stability enhance expression levels of the *Aqp1* gene during hypertonic stress [20]. Aqp protein abundance is also affected by systemic hormones, osmolality, and pH [21,22,23]. Therefore, *Aqps* are regulated by many factors, which may also reflect their extensive involvement in a variety of biological functions.

Whole genome duplication (WGD) is one of the major drivers of vertebrate evolution [24]. Two rounds of large-scale genomic duplication occurred during the early stages of vertebrate evolution [25]. The third WGD took place in fish and increased the number of *Aqp* up to 18 paralogs in zebrafish [26]. Compared to other teleosts, Atlantic salmon and common carp have undergone a fourth WGD, increasing the number of *Aqp* genes to 42 and 37, respectively [27,28]. Some duplicated genes may attain new functions through neofunctionalization and subfunctionalization or become a pseudogene after WGD [29]. For example, *Aqp7* has four pseudogenes in humans, and *Aqp10* has been lost or turned into a pseudogene in some rodents [27,30,31]. Moreover, the *Aqp3* and the *Aqp10* genes also exist as nonfunctional splice variants that lose a transmembrane domain, which may be a transitional stage in their way to becoming a pseudogene or becoming deleted. In addition, some *Aqp* members, such as *Aqp2*, *Aqp5*, and *Aqp6*, are absent in fish, suggesting that gene losses have occurred in fish ancestors after the divergence of fish and tetrapods [27,28]. Therefore, WGD and gene loss together affect the number of *Aqp* genes in organisms.

The use of organophosphorus pesticides has accelerated agricultural production over the past 30 years. Organophosphorus pesticides combine with cholinesterase on the neurosynaptic membrane, preventing its catalytic role. Thus, the accumulating acetylcholine overexcites insects and eventually causes death [32]. However, their use has also brought some pollution problems. Some aquatic organisms are seriously impacted when organophosphorus pesticides enter a water source [33]. Organophosphorus pesticides widely interfere with physiological processes, including oxidative metabolism, enzyme activities, and osmoregulation [34] Among them, oxidative metabolism induces the production of reactive oxygen species (ROS), which affects the immune response [35]. The gills and the kidney are two important osmoregulatory organs in fish. The gills participate in the exchange of electrolytes, and some contaminants, including organophosphorus pesticides, affect gill function [36]. In addition, the fish kidney is also associated with maintaining a balance of electrolytes and water. The kidney also excretes metabolic wastes, including pesticides [37]. Therefore, organophosphorus pesticides regulate the electrolyte levels of fish by affecting gill and kidney function. A previous study indicated that osmotic pressure and oxygen level can alter the expression patterns of some *Aqp* genes [38].

As an important biological resource and the first group with both innate and adaptive immune systems, studying fish not only deepens our understanding of immune system evolution in vertebrates but also helps to enhance environmental monitoring and food supply. Through a comparative evolutionary analysis, we characterized 166 putative *Aqp* genes in 12 fish species. Studies of gene loss and acquisition, gene organization, phylogeny, recombination, selective pressure, and expression patterns due to viruses, pathogenic bacteria, and organophosphorus stress were performed to explore their evolutionary relationships, which will be helpful for further functional studies of this gene family.

## 2. Materials and Methods

### 2.1. Identification of Putative Aqp Proteins in 12 Fish

The Hidden Markov Model (HMM) profile of the conserved major intrinsic protein domain (pfam00230) was used to perform a BLAST search against the Ensembl database (http://www.ensembl.org/index.html) [39] with E-value cutoff of 1E-1 to identify the putative *Aqp* genes in some fish. In addition, all Aqp paralogs reported in zebrafish [26] were used to perform another BLAST search under the same conditions. Next, all candidates identified by these searches were used for further searches. Finally, the CDD [40] and the Pfam database [41] were used to verify and confirm the reliability of these candidates. Moreover, ProtParam [42] and CELLO [43] were used to predict the biochemical characteristics and the subcellular localization of the Aqp proteins, respectively. Finally, Protter [44] was used to predict the number of transmembrane regions.

### 2.2. Estimates of Aqp Gene Gain and Loss

To determine the change in the *Aqp* gene numbers in different fish species, a gene tree and a species tree (deduced from the 28S RNA sequences) for each clade were first reconciled, and then the Notung v2.6 [45] was used to infer gene gain and loss. Notung is a reconciliation analysis software based on algorithms for tree rearrangement and reconciliation with non-binary trees. It supports the use of a parsimony-based optimization criterion to predict gene gain and loss events [45].

### 2.3. Phylogeny, Gene Organization, and Conserved Motif Analysis of the Aqp Gene Family

The MUSCLE method [46] was used to perform multiple sequence alignment of all predicted Aqp proteins. Next, MEGA6 [47] was used to construct a phylogenetic tree using the neighbor-joining method, 1000 bootstrap replications, a *p*-distance substitution model, and pairwise deletion gaps parameters. Gene organization was inferred from the Ensembl database [39]. Additionally, the MEME program [48] was used to identify conserved motifs with widths of 6 and 50, and a maximum of eight motif parameters.

### 2.4. Recombination Events and Detecting of the Aqp Genes

In this study, RDP v4.8 [49] was used to identify potential recombination events. Three methods (RDP [50], GENECONV [51], and MaxChi [52]) were used to analyze the CDS of the *Aqp* genes with a *P*-value cutoff of 0.05 and 100 permutations.

### 2.5. Site-Specific Selection Assessment and Testing

The synonymous rate (*K_s_*) and the non-synonymous rate (*K_a_*) were used to estimate selective pressure at each site. Here, we used four evolutionary models [M8 (beta+w ≥ 1), M8a (beta+w = 1), M7 (beta), and M5 (gamma)] to calculate the *K_a_/K_s_* values at each site. These models use a Bayesian inference approach to represent how the characteristics evolve with probabilistic terms [53]. Biological assumptions of different models were used to assume a statistical distribution. Next, eight discrete categories were used to approximate this distribution. Finally, the expectation of a posterior distribution was used to calculate the *K_a_/K_s_* values [53]. The Aqp protein secondary structure was predicted by Protter [44]. The Phyre2 Server [54] was used to predict the three-dimensional structure of the Dre_aqp8b protein in Group III. Finally, I-Mutant2.0 [55] was used to predict the effects of a point mutation on protein stability.

### 2.6. Microarray-Based Expression Analysis of the Zebrafish Aqp Genes under Biotic Stresses

Microarray and RNA-Seq data [56,57] were used to analyze the expression profiles of *Aqp* genes under spring viremia of carp virus (SVCV) (GSE63133) and *Mycobacterium marinum* (GSE76499) infection stress, respectively. The Genesis (v 1.7.6) program [58] was used to normalize and view their expression differences.

### 2.7. Materials, Organophosphorus Pesticide Exposure, and RNA Sequencing and Analysis

Here, we only examined the expression patterns of four fish species, including *Danio rerio*, *Oryzias latipes*, *Gasterosteus aculeatus*, and *Takifugu rubripes*, which were obtained from aquaculture farms. Each mock and experimental group consisted of six fish separately at 22–23 °C. After 2 days of environmental adaptation, an organophosphorus pesticide (dimethoate: O,O-dimethyl methylcarbamoylmethyl phosphorodithioate; C5H12NO3PS2) was added to the experimental group at a 0.92 μM concentration for 24 h. The mock group was composed of fish cultured in freshwater. In these experiments, the entire fish body was used for RNA extraction. An equimolar amount of RNA from three biological replicates was used for RNA sequencing, as described previously [59,60]. The Illumina Hiseq2000 platform was used to sequence the library at Shanghai OE Biotech Co., Ltd (Shanghai, China). The Log2 value of fragments per kilobase of transcripts per million fragments mapped (FPKM) was used to predict the expression level of each transcript. Some data were deposited at Mendeley Data [61]. The Genesis (v 1.7.6) program [58] was used to normalize the expression data.

## 3. Results and Discussion

### 3.1. Identification and Gene Gain and Loss Events of the Aqp Family Genes in the Twelve Fishes

In this study, we identified 166 putative *Aqp* genes in 12 fish species (Table S1). Each species contained 10–19 *Aqp* genes. They coded 161–336 amino acids with pI values of 4.54–10.36. In addition, 99.4% of the predicted Aqp proteins exhibited highly hydrophobic characteristics. Except the Tno_aqp4l protein, all others were predicted to be localized in the plasma membrane by CELLO [43]. Furthermore, all identified Aqp proteins had transmembrane (TM) domains. Among them, 119 Aqps (71.7%) had six TMs, consistent with their function as specific transporters of small solutes across membranes [1,2].

**Figure 1:**
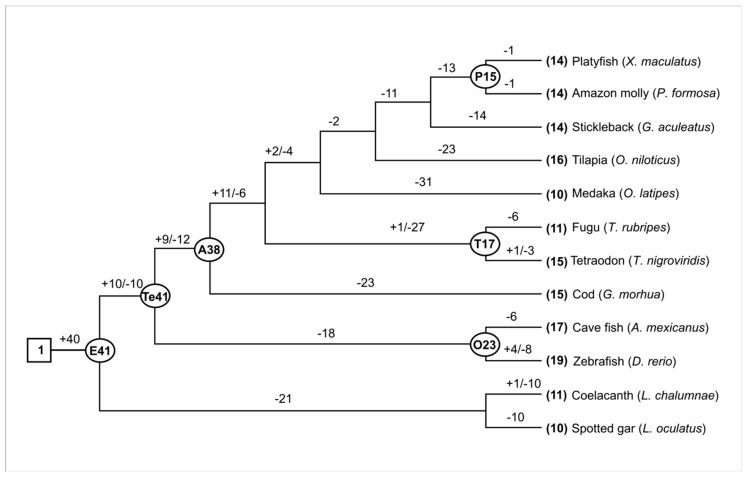
Gene gain and loss of *Aqp* in the evolution of twelve fishes. The names of internal nodes are abbreviated as E: Euteleostomi; Te: Teleostei; A: Acanthomorphata; P: Poeciliinae; T: Tetraodontidae; O: Ostariophysi. The numbers of common ancestors are shown at the internal nodes. Plus and minus signs indicate the gene gain and loss events, respectively.

Some fish, such as *D. rerio*, had 19 *Aqp* genes, while *O. latipes* and *L. oculatus* only contained 10 members, which was 47.4% lower than that of zebrafish. This finding indicates that the number of *Aqp* genes has changed dramatically in different fish species during evolution. To better understand how the *Aqp* genes evolved in these fish, we first estimated the number of *Aqp* genes in the most recent common ancestor (MRCA). One ancestral *Aqp* gene was suggested in the MRCA of these fish. Another 40 *Aqps* were obtained before the appearance of these fish (Figure 1). In total, there were about 41 ancestral *Aqp* genes in the ancestors of these fish. About 26 genes were lost and 15 were retained when the zebrafish lineage appeared. After Teleosei, the number of *Aqp* genes further changed before divergence with Acanthomorphata. We detected nine gains and 12 losses during this period, which were inherited unequally in other species (Figure 1). In general, *Aqp* gene loss was greater than gene gain in these species. For example, the numbers of lost *Aqp* genes were approximately 75.6, 65.9, 63.4, 60.9, and 53.6% for medaka, stickleback, cod, tilapia, and zebrafish, respectively (Figure 1).

### 3.2. Phylogenetic Analysis, Gene orGanization, and Motifs Distribution

**Figure 2:**
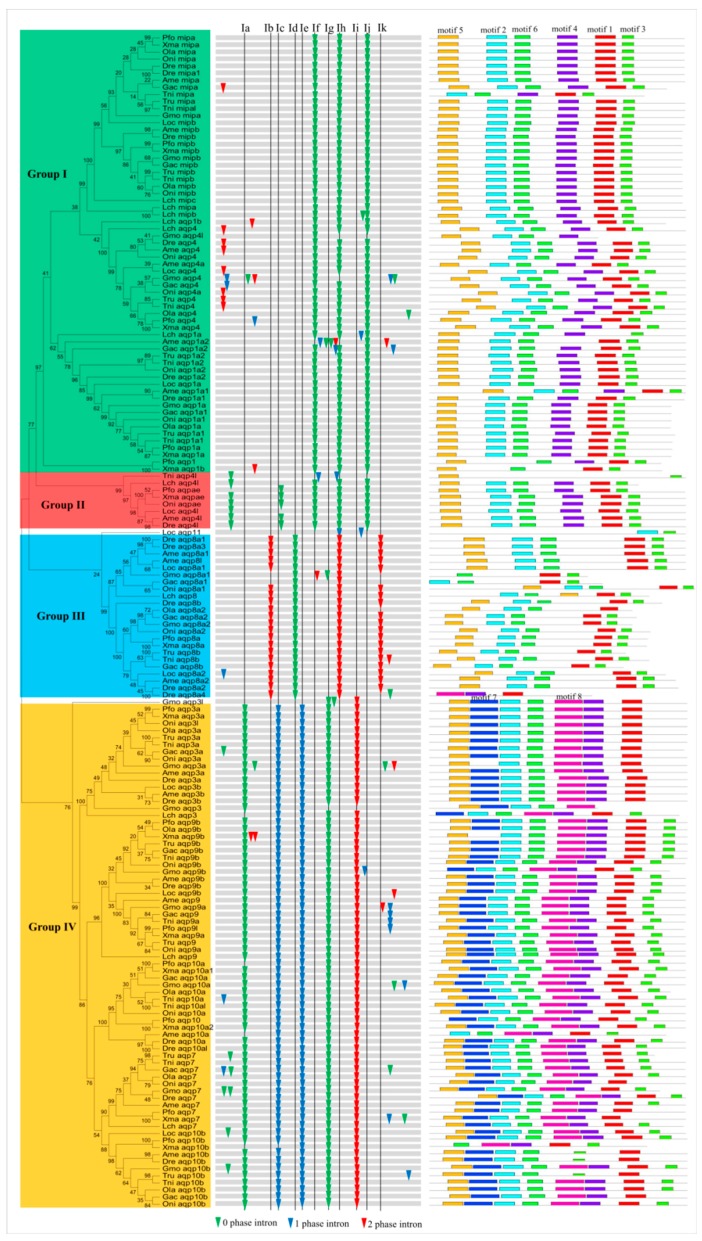
Phylogenetic relationship, gene structure, and motif composition of the *Aqp* genes in the twelve fishes. The phylogenetic tree was constructed and classified into four groups. Different motifs of the Aqp proteins are displayed by differently colored boxes. The abbreviation of species name is as follows, *Poecilia formosa*: Pfo; *Xiphophorus maculatus*: Xma; *Gasterosteus aculeatus*: Gac; *Oreochromis niloticus*: Oni; *Oryzias latipes*: Ola; *Tetraodon nigroviridis*: Tni; *Takifugu rubripes*: Tru; *Gadus morhua*: Gmo; *Danio revio*: Dre; *Astyanax mexicanus*: Ame; *Lepisosteus oculatus*: Loc; *Latimeria chalumnae*: Lch.

We carried out a phylogenetic analysis based on a neighbor-joining (NJ) method to evaluate the evolutionary relationship of *Aqp* family genes in these fish. In addition, maximum likelihood (ML) theory was used to generate another phylogenetic tree, which had a very similar topology with the NJ tree. Here, we chose the NJ tree for further analysis. The 166 Aqp proteins were divided into four families (Group I contained mipa, mipb, aqp4, and aqp1a; Group II contained aqp4l and aqpae; Group III contained aqp8a and aqp8b; and Group IV contained aqp3a, aqp3b, aqp7, aqp9a, aqp9b, aqp10a, and aqp10b) according to sequence similarity (Figure 2). Other evidence, such as gene organization and the motif distribution described below, also supported this classification. According to a previous classification [11], Groups I and II are classical aquaporins; Group III is Aqp8-type of aquaammoniaporin; and Group IV is aquaglyceroporins. These groups exhibit different functional characteristics, as described above. Moreover, aquaglyceroporins have been shown to play an important role in cellular arsenic uptake in zebrafish [62]. Group IV consists of 71 members, accounting for 42.8% of all *Aqp* genes. However, Group II contained only four genes. Moreover, we also found that some *Aqp* genes are distributed in tandem on chromosomes. For example, about 52.6% of the *Aqp* genes are linked in tandem, indicating that tandem replication contributed greatly to amplification of this gene family in fish (Table S1).

Loss or gain of introns can lead to genetic structural differences and complexity, which is the basis of the evolution of multiple gene families. Several studies have indicated that introns play important biological roles regulating gene expression, intragenic recombination, mRNA export, and exon shuffling [63,64,65]. To further study the organizational diversity of these *Aqp* genes in fish, we first compared their exon-intron structures (Figure 2). The results indicated that the exon-intron structure was well conserved in each group, suggesting a common origin. Next, we also named 11 introns from I-a to I-k to describe their loss or gain in each group. Introns I-b, I-d, and I-k existed in most of the *Aqp* genes in Group III. Insertion and phase distribution of introns I-a, I-c, I-e, I-g, and I-i were also conserved in most *Aqp* genes of Group IV. Introns I-f and I-j were specific to Group I. Interestingly, the I-h intron shared common insertion sites with Groups I, II, and III, but its phase distribution was different in Group III and the other two groups (Figure 2). In addition, some intron gain or loss events were also found in evolution, such as some non-conservative introns located in the variable domain. Thus, differences in gene structure may have triggered divergence in the *Aqp* family during fish evolution.

Next, structural diversity was compared with MEME [48], and eight conserved motifs were found in the predicted Aqp proteins (Figure 2). In general, a common motif distribution within each group existed in the majority of members, suggesting similarity in their functions. In addition, we also found several specific motifs in some groups. For example, motif 4 only occurred in Groups I and II. Motifs 7 and 8 were restricted to Group IV. We also found that two NPAs were located in motifs 2 and 1, respectively. As part of the central proton exclusion filter, the two NPAs form short hydrophobic helices and invade the membrane from the opposite direction, participating in substrate selection [3,66]. Moreover, the ar/R selectivity filter, located in conserved motif 1, has been demonstrated to determine the specificity of molecular substrates passing through the pore [5,6]. In addition to these conserved motifs, some non-conserved N- and C-terminal regions of mammalian Aqp4 are also associated with membrane trafficking and assembly [8]. Furthermore, it has been suggested that C-terminal phosphorylation (S256, S261, S264, andS269) of Aqp2 exhibits the functions of channel gating and membrane endocytosis or exocytosis [17]. Therefore, these conserved or variable regions play key roles in the functional divergence of Aqp proteins. Differences in Aqp sequences may increase the complexity of functionality.

### 3.3. Detection of Intragenic Recombination Events in the Aqp Genes

**Figure 3:**
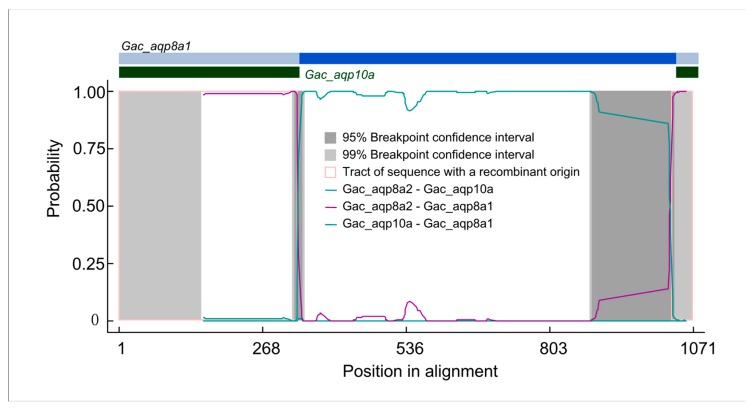
Identification of recombination events between *Gac_aqp8a1* and *Gac_aqp10a* genes. The plot display of recombination events was detected by the RDP method.

Recombination leads to intragenic sequence exchanges, which can affect gene structures and genetic variation [67,68]. To further explore the evolutionary properties of these fish *Aqp* genes, we investigated recombination events using RDP v4.8 software [49]. As summarized in Table 1, 52 *Aqp* genes were detected to contain recombination signals (*p* < 0.05 based on 100 permutations). Among them, seven *Aqp* genes in *Latimeria chalumnae* experienced recombination. For the same data, three different recombination methods gave different results. This may have been due to different assumptions made by different methods during the data generation process. A previous study indicated that the MaxChi and the GENECONV methods are more powerful than the RDP method, and that MaxChi performs the best [69]. Our results also show more recombination signals detected by the MaxChi method than the others. As an example, we presented a *Gac_aqp8a1* and a *Gac_aqp10a* recombination event detected in this study (Figure 3; Table S1). Two obvious recombination events occurred between the 5′- and the 3′-ends of *Gac_aqp8a1* and *Gac_aqp10a*, respectively. A previous study indicated that the primary structure of transmembrane helices 1–3 is similar to that of transmembrane helices 4–6 in all Aqp family proteins, suggesting that the two parts of the Aqp protein may be due to a tandem or an intragenic duplication event [70]. These results suggest that some *Aqp* genes in fish undergo frequent recombination events. Intragenic recombination increases the complexity of fish *Aqp* genes and plays an important role in the evolution of this gene family.

### 3.4. Selective Pressure at Amino Acid Sites of the Aqp Members

**Figure 4:**
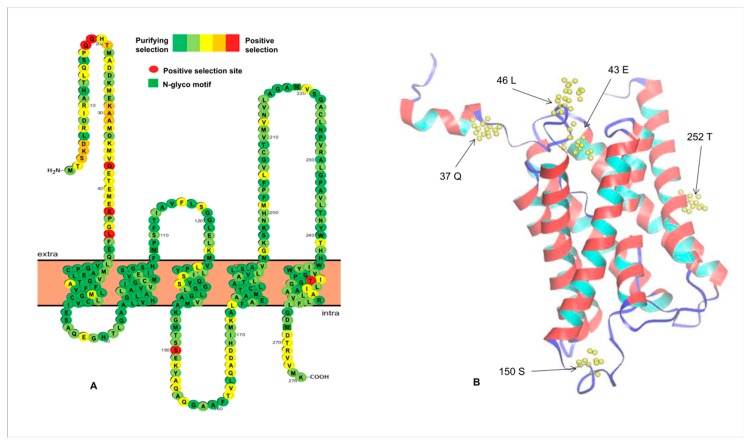
Distribution of PSS of the Group III Aqp members predicted by the M8 model. The secondary structure (**A**) and the tertiary structure (**B**) of the Dre_aqp8b protein are shown here, respectively. Since the signal peptide sequence did not appear in the three-dimensional structure of this protein, the two positive selection sites (18Q and 19Q) thereon are not labeled here.

*K_a_*/*K_s_* value was used to measure selection pressure on amino acid substitutions. This value is either greater or less than one, suggesting positive or purifying selection, respectively [71]. Different protein peptides usually undergo different selective pressures, which can further alter protein structure and function during evolution. Therefore, it is necessary to detect and analyze these residue sites under positive selection. A previous study showed that duplicated genes undergo neo-functionalization, sub-functionalization, and pseudogenization [72]. Among them, sub-functionalized and neo-functionalized genes are usually under purifying and positive selection, respectively. In this study, to explore which sites had undergone selection after duplication, the differences in the *K_a_/K_s_* values were investigated among different Aqp sites (Table 2). The *K_a_*/*K_s_* values of Group III were higher than those of the other groups, suggesting a relatively rapid change among these members. Despite these differences, all *K_a_*/*K_s_* values were below one, indicating that most fish *Aqp* genes are negatively selected during fish evolution. However, we also identified several positive selection sites in Group II and Group III (Table 2). As an example, we described, in detail, the location of these positive selection sites. There were seven sites in Group III proteins under positive selection predicted by the M8 model (Figure 4). Among them, five sites were located on the N-terminal, one on the loop, and the other on the last α-helix transmembrane domain. We did not find any positive sites on the other transmembrane helices (Figure 4). To further verify whether these positive selection sites play a role, we used I-Mutant2.0 [55] to assess their effect on protein folding stability. As a result, all mutants at these sites altered stability of the Aqp proteins (Table S2), which could facilitate functional diversification of Aqp members. Differential evolution rates of specific residues also contribute to some new functions of the *Aqp* genes after divergence. One study indicated that the *Aqp4* and the *Aqp8* genes have the longest N-termini [73]. Although the molecular function of the N-terminus is unknown in fish, its role has been associated with membrane assembly and intracellular localization in mammals [8,10] and channel gating in plants [9]. In our study, most positive sites were predicted to be located in the variable regions of the Aqp proteins, indicating functional divergence caused by the changes in these positive selection residues.

### 3.5. Expression Profiles of the Zebrafish Aqp Genes under Some Biotic Stresses

**Figure 5:**
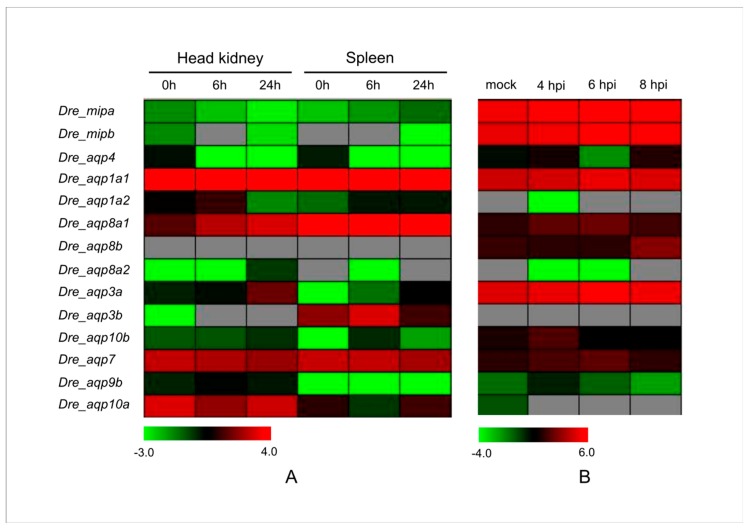
Expression profiles of the zebrafish *Aqp* genes under some biotic stresses. (**A**) Digit expression profiling of the zebrafish *Aqp* genes in the spring viremia of carp virus (SVCV)-infected tissues by microarray analysis (GSE63133). (**B**) Dynamic expression profiles of the zebrafish *Aqp* genes under the *Mycobacterium marinum* infection from GSE76499. Embryos at 4, 6, and 8 h post injection (hpi) and the uninfected mock were collected for RNA-seq analysis.

SVCV causes highly infectious viremia associated with hemorrhagic symptoms in fish [74]. An SVCV infection is highly lethal in young fish with mortality rates up to 90%, which causes huge economic losses to the aquaculture industry [75]. *Mycobacterium marinum* is a well-known pathogenic mycobacterium of skin and soft tissue infections of fish [76]. After exposure to an infected aquatic environment or animals, this pathogen can also infect humans and cause a red or tan skin bump called an aquarium granuloma [77]. To determine whether zebrafish *Aqps* are involved in the response to these biotic stressors, we further analyzed their expression patterns under SVCV and *M. marinum* infection stress via microarray and RNA-Seq data [56,57]. The results indicated that the expression levels of four *Aqp* genes were enhanced in the spleen post-SVCV infection (Figure 5). More than half of the analyzed zebrafish *Aqp* genes showed a higher expression level in the head kidney under the same stress. In addition, the *Dre_aqp3a* and the *Dre_aqp10b* transcripts increased in both SVCV infected test tissues, implying that these genes may be associated with the pathogenic response. Expression levels of more than 57% of the detected *Aqp* genes were increased after the *M. marinum* infection, and this trend was particularly evident 4 h post-injection (hpi) (Figure 5). Interestingly, *Dre_aqp8a1*, *Dre_aqp3a*, *Dre_aqp10b*, *Dre_aqp7*, and *Dre_aqp9b* genes were significantly upregulated in SVCV and *M. marinum* infected tissues, implying that these genes may be associated with the response to these biotic stressors. In addition, functional divergence of duplicated genes was also investigated. Three pairs of duplicated *Aqp* genes (*Dre_aqp1a1*/*Dre_aqp1a1*; *Dre_aqp8a2*/*Dre_aqp8b*; and *Dre_aqp3a*/*Dre_aqp3b*) (Table S1) showed different expression patterns under these stressors. For example, *M. marinum* infection induced expression of the *Dre_aqp8b* gene but decreased the transcript level of the *Dre_aqp8a2* gene. Taken together, these findings suggest that functional divergence has occurred among these duplicated genes, and that their products may play different roles in specific stress responses.

### 3.6. Expression Profiles of the Aqp Genes under Organophosphorus Pesticide Stress based on Transcriptome Data

**Figure 6:**
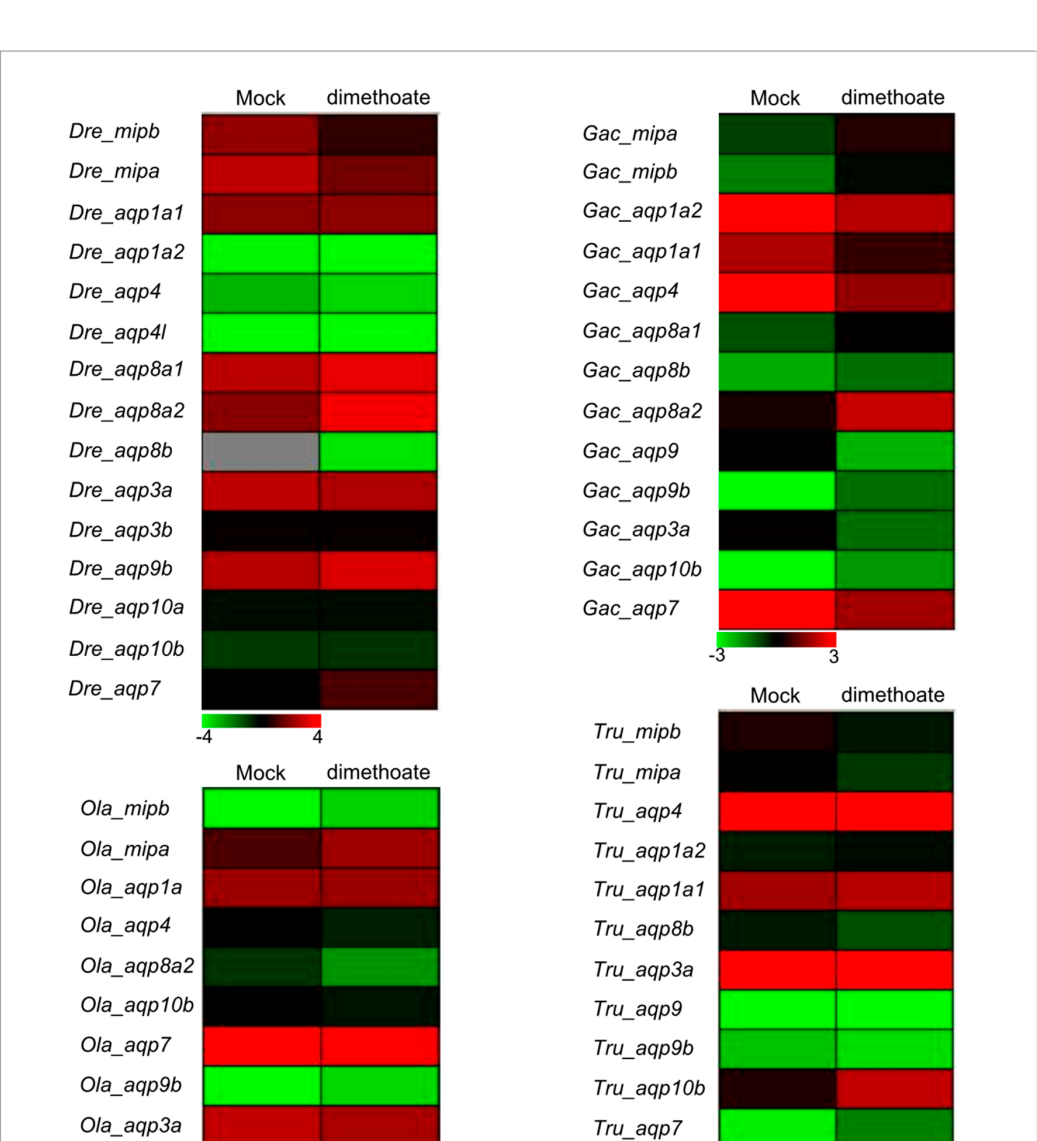
Expression profiles of zebrafish, medaka, stickleback, and fugu *Aqp* genes under dimethoate (one organophosphorus pesticide) stress based on transcriptome data.

To verify whether fish *Aqp* genes are also affected by organophosphorus pesticides, we first investigated their expression patterns under dimethoate (an organophosphorus pesticide) stress using transcriptome sequencing in four fish species, including *D. revio*, *O. latipes*, *G. aculeatus*, and *T. rubripe*. Our results indicated that expression levels of about 43.75% of the *Aqp* genes increased under dimethoate stress (Figure 6). Among them, expression levels of the *Gac_aqp8a2* and the *Gac_aqp10b* genes increased 9.7 and 7.7 times under dimethoate treatment, respectively, compared with the mock group. However, the expression level of the *Dre_mipb* gene decreased about 4.2 times under the same conditions. The response of some duplicated *Aqp* genes (such as *Dre_aqp1a1*/*Dre_aqp1a2*, *Gac_aqp9*/*Gac_aqp9b*, and *Ola_mipa*/*Ola_mipb*) (Table S1) to dimethoate treatment was also significantly different (Figure 6). As a group of water channel proteins, Aqps are involved in many physiological functions and pathological processes, such as transepithelial transport [78], tumorigenesis [79], cell migration [80], and neuronal signal transduction [81]. Furthermore, organophosphorus pesticides have been demonstrated to affect immunoregulation in fish [82]. Here, the expression levels of some fish *Aqp* genes were induced under dimethoate stress, suggesting that these *Aqps* may be involved in fish immunoregulation.

## 4. Conclusions

A comparative analysis of the fish *Aqp* gene family was performed in this study, which was divided into four groups by phylogenetic analyses. Gene organization and motif distribution were highly conserved in each group, implying their functional relevance. During the evolution process, some fish *Aqp* genes have experienced intragenic recombination. Selection pressure analysis identified several sites that might be associated with functional divergence. Differential expression profiles of some *Aqp* genes under SVCV and *M. marinum* infection and dimethoate stress provided insights into possible functions. This study can provide a useful reference for further functional study.

## Figures and Tables

**Table 1:** The predicted recombination events of the *Aqp* genes in the twelve fishes.

Species	Recombination Methods			Genes undergone Recombination Events
	RDP	GENECONV	MaxChi	
*A. mexicanus*	1	1	4	*Ame_aqp3a; Ame_aqp7; Ame_aqp8a1; Ame_aqp8a2; Ame_aqp10b; Ame_mipa*
*D. rerio*	1	2	5	*Dre_aqp3a; Dre_aqp3*
*G. aculeatus*	3	0	4	*Gac_aqp4; Gac_aqp8a1; Gac_aqp9b; Gac_aqp10a*
*G. morhua*	1	0	2	*Gmo_aqp1a; Gmo_aqp10b*
*L. chalumnae*	2	0	3	*Lch_aqp3; Lch_aqp4; Lch_aqp4l; Lch_aqp7; Lch_aqp9; Lch_mipb; Lch_mipc*
*L. oculatus*	2	0	1	*Loc_aqp3b; Loc_aqp9b; Loc_aqp10b; Loc_mipb*
*O. latipes*	1	0	5	*Ola_aqp4; Ola_aqp9b; Ola_10b; Ola_mipa*
*O. niloticus*	1	0	2	*Oni_aqp4; Oni_aqp8a1; Oni_aqp9a*
*P. formosa*	4	0	4	*Pfo_aqp1; Pfo_aqp9l; Pfo_aqp9b; Pfo_aqp10; Pfo_aqp10a; Pfo_mipb*
*T. nigroviridis*	4	1	6	*Tni_aqp3a; Tni_aqp4; Tni_aqp7; Tni_aqp9b*
*T. rubripes*	3	0	5	*Tru_aqp1a1; Tru_aqp3a; Tru_aqp7; Tru_aqp9*
*X. maculatus*	2	0	6	*Xma_aqp1a; Xma_aqp3a; Xma_aqp7; Xma_aqp8a; Xma_aqp9b; Xma_10a2*

**Table 2:** Likelihood values and parameter estimates of the *Aqp* genes.

Gene Branches	Selection Models	*K_a_/K_s_*	Log-Likehood	Numbers of PSS *
Group I	M8(beta+w ≥ 1)	0.2236	−29,922.3	0
	M8a(beta+w = 1)	0.2222	−29,924.8	0
	M7(beta)	0.2069	−29,913.2	0
	M5(gamma)	0.2279	−29,941	0
Group II	M8(beta+w ≥ 1)	0.2219	−5268.97	0
	M8a(beta+w = 1)	0.2185	−5267.87	0
	M7(beta)	0.2165	−5265.73	0
	M5(gamma)	0.2403	−5279.27	0
Group III	M8(beta+w ≥ 1)	0.2628	−11,574.5	7
	M8a(beta+w = 1)	0.2567	−11,572.8	0
	M7(beta)	0.2521	−11,580.1	0
	M5(gamma)	0.2719	−11,582.4	0
Group IV	M8(beta+w ≥ 1)	0.2141	−37,259.4	5
	M8a(beta+w = 1)	0.2096	−37,265.2	0
	M7(beta)	0.2004	−37,281.1	0
	M5(gamma)	0.2120	−37,277.5	0

* PSS: positive selection site.

## Supplementary Materials

The following are available online at https://www.mdpi.com/2076-2615/9/5/233/s1, Table S1: Aqp genes identified in the twelve fishes, Table S2: Effect of positive selection site mutation on the stability of Dre_aqp8b protein.

## References

[1] Agre P., Preston G.M., Smith B.L., Jung J.S., Raina S., Moon C., Guggino W.B., Nielsen S. (1993). Aquaporin CHIP: The archetypal molecular water channel. Am. J. Physiol..

[2] Heymann J.B., Engel A. (1999). Aquaporins: Phylogeny, Structure, and Physiology of Water Channels. News Physiol. Sci..

[3] Ikeda M., Andoo A., Shimono M., Takamatsu N., Taki A., Muta K., Matsushita W., Uechi T., Matsuzaki T., Kenmochi N. (2011). The NPC motif of aquaporin-11, unlike the NPA motif of known aquaporins, is essential for full expression of molecular function. J. Biol. Chem..

[4] Takata K., Matsuzaki T., Tajika Y. (2004). Aquaporins: Water channel proteins of the cell membrane. Prog. Histochem. Cytochem..

[5] Murata K., Mitsuoka K., Hirai T., Walz T., Agre P., Heymann J.B., Engel A., Fujiyoshi Y. (2000). Structural determinants of water permeation through aquaporin-1. Nature.

[6] Sui H., Han B.G., Lee J.K., Walian P., Jap B.K. (2001). Structural basis of water-specific transport through the AQP1 water channel. Nature.

[7] Madsen S.S., Engelund M.B., Cutler C.P. (2015). Water transport and functional dynamics of aquaporins in osmoregulatory organs of fishes. Biol. Bull..

[8] Neely J.D., Christensen B.M., Nielsen S., Agre P. (1999). Heterotetrameric composition of aquaporin-4 water channels. Biochemistry.

[9] Törnroth-Horsefield S., Wang Y., Hedfalk K., Johanson U., Karlsson M., Tajkhorshid E., Neutze R., Kjellbom P. (2006). Structural mechanism of plant aquaporin gating. Nature.

[10] Beitz E., Liu K., Ikeda M., Guggino W.B., Agre P., Yasui M. (2006). Determinants of AQP6 trafficking to intracellular sites versus the plasma membrane in transfected mammalian cells. Biol. Cell.

[11] Finn R.N., Cerdà J. (2015). Evolution and functional diversity of aquaporins. Biol. Bull..

[12] Ishibashi K., Morishita Y., Tanaka Y. (2017). The Evolutionary Aspects of Aquaporin Family. Adv. Exp. Med. Biol..

[13] Jung S.Y., Kim S.S., Kim Y.I., Kim S.H., Yeo S.G. (2017). A Review: Expression of Aquaporins in Otitis Media. Int. J. Mol. Sci..

[14] Cerdà J., Finn R.N. (2010). Piscine aquaporins: An overview of recent advances. J. Exp. Zool. A Ecol. Genet. Physiol..

[15] Froger A., Clemens D., Kalman K., Németh-Cahalan K.L., Schilling T.F., Hall J.E. (2010). Two distinct aquaporin 0s required for development and transparency of the zebrafish lens. Invest. Ophthalmol. Vis. Sci..

[16] Koun S., Kim J.D., Rhee M., Kim M.J., Huh T.L. (2016). Spatiotemporal expression pattern of the zebrafish *aquaporin 8 family* during early developmental stages. Gene Expr. Patterns.

[17] Moeller H.B., Olesen E.T., Fenton R.A. (2011). Regulation of the water channel aquaporin-2 by posttranslational modification. Am. J. Physiol. Renal Physiol..

[18] Chauvigné F., Boj M., Finn R.N., Cerdà J. (2015). Mitochondrial aquaporin-8-mediated hydrogen peroxide transport is essential for teleost spermatozoon motility. Sci. Rep..

[19] Fushimi K., Sasaki S., Marumo F. (1997). Phosphorylation of serine 256 is required for cAMP-dependent regulatory exocytosis of the aquaporin-2 water channel. J. Biol. Chem..

[20] Leitch V., Agre P., King L.S. (2001). Altered ubiquitination and stability of aquaporin-1 in hypertonic stress. Proc. Natl. Acad. Sci. USA.

[21] Catalán V., Gómez-Ambrosi J., Pastor C., Rotellar F., Silva C., Rodríguez A., Gil M.J., Cienfuegos J.A., Salvador J., Vendrell J., Frühbeck G. (2008). Influence of morbid obesity and insulin resistance on gene expression levels of AQP7 in visceral adipose tissue and AQP9 in liver. Obes. Surg..

[22] Umenishi F., Schrier R.W. (2003). Hypertonicity-induced aquaporin-1 (AQP1) expression is mediated by the activation of MAPK pathways and hypertonicity-responsive element in the AQP1 gene. J. Biol. Chem..

[23] Choi H.J., Jung H.J., Kwon T.H. (2015). Extracellular pH affects phosphorylation and intracellular trafficking of AQP2 in inner medullary collecting duct cells. Am. J. Physiol. Renal Physiol..

[24] Brunet F.G., Roest Crollius H., Paris M., Aury J.M., Gibert P., Jaillon O., Laudet V., Robinson-Rechavi M. (2006). Gene loss and evolutionary rates following whole-genome duplication in teleost fishes. Mol. Biol. Evol..

[25] Hughes T., Liberles D.A. (2008). Whole-genome duplications in the ancestral vertebrate are detectable in the distribution of gene family sizes of tetrapod species. J. Mol. Evol..

[26] Tingaud-Sequeira A., Calusinska M., Finn R.N., Chauvigné F., Lozano J., Cerdà J. (2010). The zebrafish genome encodes the largest vertebrate repertoire of functional aquaporins with dual paralogy and substrate specificities similar to mammals. BMC Evol. Biol..

[27] Finn R.N., Chauvigné F., Hlidberg J.B., Cutler C.P., Cerdà J. (2014). The lineage-specific evolution of aquaporin gene clusters facilitated tetrapod terrestrial adaptation. PLoS ONE.

[28] Dong C., Chen L., Feng J., Xu J., Mahboob S., Al-Ghanim K., Li X., Xu P. (2016). Genome Wide Identification, Phylogeny, and Expression of Aquaporin Genes in Common Carp (*Cyprinus carpio*). PLoS ONE.

[29] Sémon M., Wolfe K.H. (2007). Consequences of genome duplication. Curr. Opin. Genet. Dev..

[30] Morinaga T., Nakakoshi M., Hirao A., Imai M., Ishibashi K. (2002). Mouse aquaporin 10 gene (AQP10) is a pseudogene. Biochem. Biophys. Res. Commun..

[31] Tanaka Y., Morishita Y., Ishibashi K. (2015). Aquaporin10 is a pseudogene in cattle and their relatives. Biochem. Biophys. Rep..

[32] Kwong T.C. (2002). Organophosphate pesticides: Biochemistry and clinical toxicology. Ther. Drug Monit..

[33] Fulton M.H., Key P.B. (2001). Acetylcholinesterase inhibition in estuarine fish and invertebrates as an indicator of organophosphorus insecticide exposure and effects. Environ. Toxicol. Chem..

[34] Yeh S.P., Sung T.G., Chang C.C., Chen W., Kuo C.M. (2005). Effects of an organophosphorus insecticide, trichlorfon, on hematological parameters of the giant freshwater prawn, *Macrobrachium rosenbergii* (de Man). Aquaculture.

[35] Yang Y., Bazhin A.V., Werner J., Karakhanova S. (2013). Reactive oxygen species in the immune system. Int. Rev. Immunol..

[36] Wendelaar Bonga S., Lock R. (1991). Toxicants and osmoregulation in fish. Neth. J. Zool..

[37] Katuli K.K., Amiri B.M., Massarsky A., Yelghi S., Ghasemzadeh J. (2014). Impact of a short-term diazinon exposure on the osmoregulation potentiality of Caspian roach (*Rutilus rutilus*) fingerlings. Chemosphere.

[38] Yamamoto N., Yoneda K., Asai K., Sobue K., Tada T., Fujita Y., Katsuya H., Fujita M., Aihara N., Mase M., Yamada K., Miura Y., Kato T. (2001). Alterations in the expression of the AQP family in cultured rat astrocytes during hypoxia and reoxygenation. Brain Res. Mol. Brain Res..

[39] Herrero J., Muffato M., Beal K., Fitzgerald S., Gordon L., Pignatelli M., Vilella A.J., Searle S.M., Amode R., Brent S., Spooner W., Kulesha E., Yates A., Flicek P. (2016). Ensembl comparative genomics resources. Database.

[40] Marchler-Bauer A., Derbyshire M.K., Gonzales N.R., Lu S., Chitsaz F., Geer L.Y., Geer R.C., He J., Gwadz M., Hurwitz D.I. (2015). CDD: NCBI’s conserved domain database. Nucleic Acids Res..

[41] Punta M., Coggill P.C., Eberhardt R.Y., Mistry J., Tate J., Boursnell C., Pang N., Forslund K., Ceric G., Clements J. (2012). The Pfam protein families database. Nucleic Acids Res..

[42] Wilkins M.R., Gasteiger E., Bairoch A., Sanchez J.C., Williams K.L., Appel R.D., Hochstrasser D.F. (1999). Protein identification and analysis tools in the ExPASy server. Methods Mol. Biol..

[43] Yu C.S., Lin C.J., Hwang J.K. (2004). Predicting subcellular localization of proteins fro Gram-negative bacteria by support vector machines based on n-peptide compositions. Protein Sci..

[44] Omasits U., Ahrens C.H., Müller S., Wollscheid B. (2014). Protter: Interactive protein feature visualization and integration with experimental proteomic data. Bioinformatics.

[45] Chen K., Durand D., Farach-Colton M. (2000). NOTUNG: A program for dating gene duplications and optimizing gene family trees. J. Comput. Biol..

[46] Edgar R.C. (2004). MUSCLE: A multiple sequence alignment method with reduced time and space complexity. BMC Bioinform..

[47] Tamura K., Stecher G., Peterson D., Filipski A., Kumar S. (2013). MEGA6: Molecular Evolutionary Genetics Analysis version 6.0. Mol. Biol. Evol..

[48] Bailey T.L., Williams N., Misleh C., Li W.W. (2006). MEME: discovering and analyzing DNA and protein sequence motifs. Nucleic Acids Res..

[49] Martin D.P., Lemey P., Lott M., Moulton V., Posada D., Lefeuvre P. (2010). RDP3: A flexible and fast computer program for analyzing recombination. Bioinformatics.

[50] Martin D., Rybicki E. (2000). RDP: detection of recombination amongst aligned sequences. Bioinformatics.

[51] Padidam M., Sawyer S., Fauquet C.M. (1999). Possible emergence of new geminiviruses by frequent recombination. Virology.

[52] Posada D., Crandall K.A. (2001). Evaluation of methods for detecting recombination from DNA sequences: computer simulations. Proc. Natl. Acad. Sci. USA.

[53] Stern A., Doron-Faigenboim A., Erez E., Martz E., Bacharach E., Pupko T. (2007). Selecton 2007: Advanced models for detecting positive and purifying selection using a Bayesian inference approach. Nucleic Acids Res..

[54] Kelley L.A., Mezulis S., Yates C.M., Wass M.N., Sternberg M.J. (2015). The Phyre2 web portal for protein modeling, prediction and analysis. Nat. Protoc..

[55] Capriotti E., Fariselli P., Casadio R. (2005). I-Mutant2.0: Predicting stability changes upon mutation from the protein sequence or structure. Nucleic Acids Res..

[56] Feng H., Zhang Y.B., Zhang Q.M., Li Z., Zhang Q.Y., Gui J.F. (2015). Zebrafish IRF1 regulates IFN antiviral response through binding to IFNϕ1 and IFNϕ3 promoters downstream of MyD88 signaling. J. Immunol..

[57] Benard E.L., Rougeot J., Racz P.I., Spaink H.P., Meijer A.H. (2016). Transcriptomic Approaches in the Zebrafish Model for Tuberculosis-Insights Into Host- and Pathogen-specific Determinants of the Innate Immune Response. Adv. Genet..

[58] Sturn A., Quackenbush J., Trajanoski Z. (2002). Genesis: Cluster analysis of microarray data. Bioinformatics.

[59] Cao J., Tan X. (2018). Comparative and evolutionary analysis of the 14-3-3 family genes in eleven fishes. Gene.

[60] Cao J., Tan X. (2018). Comparative analysis of the *tetraspanin* gene family in six teleost fishes. Fish Shellfish Immunol..

[61] Mendeley Data. https://data.mendeley.com/datasets/fch4sczvx7/1.

[62] Hamdi M., Sanchez M.A., Beene L.C., Liu Q., Landfear S.M., Rosen B.P., Liu Z. (2009). Arsenic transport by zebrafish aquaglyceroporins. BMC Mol. Biol..

[63] Castillo-Davis C.I., Mekhedov S.L., Hartl D.L., Koonin E.V., Kondrashov F.A. (2002). Selection for short introns in highly expressed genes. Nat. Genet..

[64] Le Hir H., Nott A., Moore M.J. (2003). How introns influence and enhance eukaryotic gene expression. Trends Biochem. Sci..

[65] Roy S.W., Gilbert W. (2006). The evolution of spliceosomal introns: Patterns, puzzles and progress. Nat. Rev. Genet..

[66] Gomes D., Agasse A., Thiébaud P., Delrot S., Gerós H., Chaumont F. (2009). Aquaporins are multifunctional water and solute transporters highly divergent in living organisms. Biochim. Biophys. Acta.

[67] de Silva E., Kelley L.A., Stumpf M.P. (2004). The extent and importance of intragenic recombination. Hum. Genomics.

[68] Wicker T., Yahiaoui N., Keller B. (2007). Illegitimate recombination is a major evolutionary mechanism for initiating size variation in plant resistance genes. Plant J..

[69] Posada D. (2002). Evaluation of methods for detecting recombination from DNA sequences: Empirical data. Mol. Biol. Evol..

[70] Reizer J., Reizer A., Saier M.H. (1993). The MIP family of integral membrane channel proteins: Sequence comparisons, evolutionary relationships, reconstructed pathway of evolution, and proposed functional differentiation of the two repeated halves of the proteins. Cri. Rev. Biochem. Mol. Biol..

[71] Hurst L.D. (2002). The *Ka*/*Ks* ratio: Diagnosing the form of sequence evolution. Trends Genet..

[72] Duarte J.M., Cui L., Wall P.K., Zhang Q., Zhang X., Leebens-Mack J., Ma H., Altman N., dePamphilis C.W. (2006). Expression pattern shifts following duplication indicative of subfunctionalization and neofunctionalization in regulatory genes of Arabidopsis. Mol. Biol. Evol..

[73] Finn R.N., Cerdà J. (2011). Aquaporin evolution in fishes. Front. Physiol..

[74] Ahne W., Bjorklund H.V., Essbauer S., Fijan N., Kurath G., Winton J.R. (2002). Spring viremia of carp (SVC). Dis. Aquat. Organ..

[75] Ashraf U., Lu Y., Lin L., Yuan J., Wang M., Liu X. (2016). Spring viraemia of carp virus: Recent advances. J. Gen. Virol..

[76] Chan K., Knaak T., Satkamp L., Humbert O., Falkow S., Ramakrishnan L. (2002). Complex pattern of *Mycobacterium marinum* gene expression during long-term granulomatous infection. Proc. Natl. Acad. Sci. USA.

[77] Wu T.S., Chiu C.H., Yang C.H., Leu H.S., Huang C.T., Chen Y.C., Wu T.L., Chang P.Y., Su L.H., Kuo A.J., Chia J.H., Lu C.C., Lai H.C. (2012). Fish tank granuloma caused by *Mycobacterium marinum*. PLoS ONE.

[78] Gonen T., Walz T. (2006). The structure of aquaporins. Q. Rev. Biophys..

[79] Saadoun S., Papadopoulos M.C., Hara-Chikuma M., Verkman A.S. (2005). Impairment of angiogenesis and cell migration by targeted aquaporin-1 gene disruption. Nature.

[80] Saadoun S., Papadopoulos M.C., Watanabe H., Yan D., Manley G.T., Verkman A.S. (2005). Involvement of aquaporin-4 in astroglial cell migration and glial scar formation. J. Cell Sci..

[81] Da T., Verkman A.S. (2004). Aquaporin-4 gene disruption in mice protects against impaired retinal function and cell death after ischemia. Invest. Ophthalmol. Vis. Sci..

[82] Díaz-Resendiz K.J., Toledo-Ibarra G.A., Girón-Pérez M.I. (2015). Modulation of immune response by organophosphorus pesticides: Fishes as a potential model in immunotoxicology. J. Immunol. Res..

